# Requirement for written informed consent and selection bias in a chart-review prostate cancer study

**DOI:** 10.1093/aje/kwag043

**Published:** 2026-03-04

**Authors:** Renning Zheng, Sanjay K Das, Trung Duong Tran, Nadine A Friedrich, Stirling M Cummings, Anakaren Gonzalez, Amanda M De Hoedt, Haleigh Bellerose, Anna Hoffmeyer, Thomas J Van de Ven, Stephen J Freedland

**Affiliations:** Department of Urology, Cedars-Sinai Medical Center, Los Angeles, CA, United States; Tsinghua Medicine, Tsinghua University, Beijing, China; Department of Urology, Cedars-Sinai Medical Center, Los Angeles, CA, United States; Cleveland Clinic, Digestive Disease and Surgery Institute, Cleveland, OH, United States; Department of Surgery, Durham Veterans Affairs Health Care System, Durham, NC, United States; Department of Urology, Cedars-Sinai Medical Center, Los Angeles, CA, United States; Department of Surgery, Durham Veterans Affairs Health Care System, Durham, NC, United States; Department of Urology, Cedars-Sinai Medical Center, Los Angeles, CA, United States; College of Health and Human Services, California State University, Long Beach, Long Beach, CA, United States; Department of Surgery, Durham Veterans Affairs Health Care System, Durham, NC, United States; Department of Surgery, Durham Veterans Affairs Health Care System, Durham, NC, United States; Department of Surgery, Durham Veterans Affairs Health Care System, Durham, NC, United States; Institutional Review Board, Durham Veterans Affairs Health Care System, Durham, NC, United States; Department of Anesthesiology, Duke University School of Medicine and the Durham Veterans Affairs Health Systems, Durham, NC, United States; Department of Urology, Cedars-Sinai Medical Center, Los Angeles, CA, United States; Department of Surgery, Durham Veterans Affairs Health Care System, Durham, NC, United States; Samuel Oschin Comprehensive Cancer Institute, Cedars-Sinai Medical Center, Los Angeles, CA, United States

**Keywords:** selection bias, informed consent, chart-review study, prospective enrollment

## Abstract

Although retrospective chart-review studies are typically performed using a waiver of written informed consent, many institutional review boards (IRB) do not approve such waivers for chart-review studies using prospective enrollment, which could introduce selection bias in participant characteristics and outcomes, thereby impairing representativeness and validity. We aim to determine this bias in a chart-review prostate cancer (PCa) study using prospective enrollment. Using an IRB-approved chart-review protocol with a waiver of written informed consent for prospective enrollment, we identified 2202 patients scheduled for initial prostate biopsy from 2007 to 2021 at Durham Veterans Affairs Healthcare System. These patients were simultaneously approached for enrollment into a separate minimal-risk prospective observational study protocol requiring blood collection and written consent. One thousand two hundred thirty-eight subjects provided written consent to the blood collection protocol; 964 did not. Patients who provided written consent differed in several key characteristics, including younger age, but had a similar racial distribution. Importantly, participants providing written consent had a significantly lower risk of PCa (multivariable OR = 0.41; 95% CI, 0.31-0.54; *P* < .001). As such, patients who provided written consent had younger age, similar race, and lower PCa risk and therefore might not accurately represent the full eligible population. To minimize selection bias, a waiver of written consent should be allowed for chart-review studies using prospective enrollment.

## Introduction

Informed consent is a fundamental requirement in most studies involving interaction with participants as it ensures that patients are fully aware of the risks and benefits of participation. However, regulations allow institutional review boards (IRB) to waive the requirement for written consent if the research involves no more than minimal risk (eg, chart review only), it could not practicably be carried out without the waiver, and participants can be provided with further information about the study when appropriate.^1^ This is because that minimal-risk studies do not interfere with clinical practice, and it is often impractical to obtain written consent from all patients. While waivers of written informed consent for retrospective chart-review analyses are relatively standard, not all IRBs will approve the waiver of informed consent when prospective enrollment is used.^2^^-^^5^ In cases where use of the written informed consent waiver for prospective enrollment is not allowed, study follow-up over time can only be updated when written consent is obtained from participants.

A significant concern regarding the requirement for written informed consent is the potential for selection bias, as participants who provide written consent might not accurately represent the full eligible population.^6^ However, such bias would be avoided in chart-review studies with prospective enrollment where a waiver of written informed consent may be permitted due to its minimal-risk nature. While such bias is seldom quantified, it might seriously compromise the accuracy and validity of the study. For example, when establishing the Registry of Canadian Stroke Network, researchers found that patients who consented to enrollment had different baseline characteristics and significantly lower mortality rates compared to those who did not.^7^ In response, the researchers eliminated the written consent requirement and used de-identified data with a waiver of written informed consent instead to obtain a more representative sample.^7^ Therefore, estimating the extent of selection bias due to the requirement for written consent is crucial for understanding how requiring written consent in the prospective enrollment of chart-review research can influence the conclusions drawn from the study.

The effect of selection bias due to requiring written consent in prospective enrollment of chart-review studies was further demonstrated in a systematic review of 17 studies. This review found significant differences in demographic characteristics such as age, sex, and race between participants and nonparticipants prospectively approached for consent for the use of their medical records.^8^ However, the direction and magnitude of these differences varied considerably depending on the focus of the individual studies. For example, among 15 studies reporting the age of participants and nonparticipants, 7 found no differences, 1 found that participants were younger, and 7 identified significant differences across age strata. Additionally, only 6 of these studies assessed differences in patient prognoses or primary study outcomes beyond demographics. While a more recent study found that breast cancer patients who consented to inclusion into a registry had better prognoses than those who did not,^9^ the investigation on implications of written informed consent on clinical results and study outcomes remain largely unexplored.

Prostate cancer (PCa) is the most common cancer and the second leading cause of cancer-related mortality among men in the United States.^10^ Although numerous studies have been conducted to improve PCa management,^11^ none have specifically examined the selection bias due to requiring written consent. In this study, we had a unique opportunity to evaluate this bias among participants in a chart-review study using prospective enrollment with a waiver of written informed consent. We compared the characteristics and PCa risk by whether participants provided written consent (or not) to another separate minimal-risk prospective observational study protocol that required blood collection. Additionally, it is well established that Black patients have a higher risk of PCa. Beyond genetic factors, other influences, including cultural, social, and economic factors, may also contribute to this disparity.^12^ Given that race, potentially through these underlying socioeconomic factors, may affect both consent status and PCa risk,^8^^,^^12^ it represents an important potential confounder. Accordingly, we performed a secondary analysis stratified by race to account for this effect. Moreover, these analyses test the clinical relevance of the selection bias from requiring written consent and thus test whether the end result is different conclusions regarding a well-known PCa risk factor.

## Methods

### Study population

This was a nonrandomized, real-world observational study in which demographic characteristics, consent status, and study outcomes were determined by two independent study protocols. After obtaining approval from the IRB of Durham Veterans Affairs Health Care System (DVAHCS), we conducted a chart-review study involving all patients undergoing prostate biopsy at the DVAHCS. Per our IRB approval, we used prospective enrollment with a waiver of written informed consent due to the impracticality of obtaining written consent for all patients and the minimal-risk nature of the chart-review study.

Meanwhile, from January 2007 to June 2012 and again from July 2013 to December 2021, patients scheduled for prostate biopsy at the DVAHCS were approached for enrollment into a separate minimal-risk prospective observational study approved by the IRB of DVAHCS. The details of the study have been previously described.^13^^-^^15^ In brief, patients providing written consent gave blood samples and completed detailed questionnaires beyond routine clinical care. As such, written consent was necessary to collect the questionnaire data and samples.

**Figure 1:**
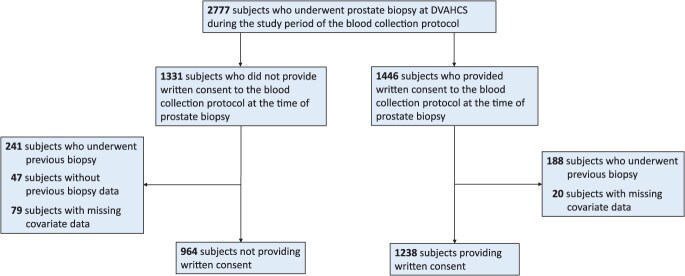
Consort diagram of the study population. Abbreviation: DVAHCS, Durham Veterans Affairs Health Care System.

Per protocol of the chart-review only study, we identified 2777 patients scheduled for prostate biopsies at the DVAHCS during the study period of the blood collection protocol, among whom 1331 subjects did not provide written consent to the blood collection protocol and 1446 provided written consent to the blood collection protocol. Patients not providing written consent to the blood collection protocol were studied using the waiver of written informed consent protocol. To ensure consistent biopsy results across both cohorts, only the patient’s initial biopsy was included in the analysis. Therefore, we excluded 476 patients who had undergone previous biopsy or without previous biopsy data. Additionally, 99 participants with incomplete covariate data were also excluded. The final study population consisted of 2202 participants, including 964 (44%) subjects not providing written consent and 1238 (56%) subjects providing written consent (Figure 1).

### Statistical analysis

Patient baseline demographic and clinical characteristics were summarized and compared using the Wilcoxon rank-sum test for continuous variables and the chi-square test for categorical variables. A multivariable logistic regression model was used to assess the association between baseline characteristics and written consent status, adjusting for other covariates.

PCa status (yes/no) was determined based on the results of the biopsy. We first used a multivariable logistic regression model to estimate the association between written consent status and overall PCa outcome (yes/no). As patients with low-grade PCa (grade group 1) and high-grade PCa (grade groups 2–5) differ in risk factors, clinical features, and treatment, we additionally employed stratified multinomial logistic regression models to assess whether associations varied by PCa grade (low- and high-grade PCa relative to no cancer), adjusted for age at biopsy (continuous), race (non-Black, Black), year of biopsy (continuous), prostate-specific antigen (PSA) (continuous, ng/mL, log-transformed), digital rectal exam (DRE) (not suspicious for cancer, suspicious for cancer), and prostate volume (continuous, cc, log-transformed). To investigate the potential interaction between race and written consent status in predicting PCa (yes/no) and PCa grade (low- or high-grade vs no cancer), we introduced and tested the interaction term in the models and performed secondary analyses stratified by race.

## Results

Compared to patients not providing written consent, those providing written consent were slightly younger, received prostate biopsy more recently, and had lower PSA levels, slightly larger prostates, and a lower likelihood of being diagnosed with PCa. No differences were observed in race or DRE results (Table 1).

**Table 1:** Baseline characteristics of participants by written consent status

**Variable**	**Subjects not providing written consent (*n* = 964)**	**Subjects providing written consent (*n* = 1238)**	***P***
Age at biopsy, median (Q1, Q3)	64 (60, 69)	64 (60, 68)	.017^a^
Race			.490^b^
Non-Black	380 (39%)	506 (41%)	
Black	584 (61%)	732 (59%)	
Year of biopsy, median (Q1, Q3)	2010 (2008, 2016)	2013 (2009, 2017)	<.001^a^
PSA at biopsy (ng/mL), median (Q1, Q3)	6.2 (4.8, 9.9)	5.7 (4.5, 8.0)	<.001^a^
DRE			.472^b^
Not suspicious for cancer	681 (71%)	857 (69%)	
Suspicious for cancer	283 (29%)	381 (31%)	
Prostate volume (cc), median (Q1, Q3)	37.0 (27.0, 52.0)	39.8 (28.0, 54.0)	.031^a^
Prostate cancer status			<.001^b^
No cancer	295 (31%)	518 (42%)	
Low-grade cancer	240 (25%)	298 (24%)	
High-grade cancer	429 (45%)	422 (34%)	

Abbreviations: DRE, digital rectal exam; PSA, prostate-specific antigen.

^a^Wilcoxon rank sum test.

^b^Chi-square test.

In multivariable analysis, consistent with unadjusted results, older patients and those with higher PSA levels were less likely to provide written consent, whereas those with recent biopsies or larger prostates were more likely to provide written consent. Race and DRE remained unassociated with written consent status (Table 2).

**Table 2:** Multivariable logistic regression models assessing the association between baseline characteristics and the likelihood of providing written consent.

**Variable**	**Providing written consent** ^**a**^	
	**OR (95% CI)**	***P***
Age at biopsy (unit = 1 year)	0.97 (0.96—0.98)	<.001
Race		
Non-Black	ref	
Black	0.89 (0.74—1.07)	.230
Year of biopsy (unit = 1 year)	1.08 (1.06—1.11)	<.001
PSA (ng/mL, log-transformed)	0.74 (0.67—0.81)	<.001
DRE		
Not suspicious for cancer	ref	
Suspicious for cancer	1.19 (0.98—1.44)	.082
Prostate volume (cc, log-transformed)	1.32 (1.10—1.58)	.003

Abbreviations: DRE, digital rectal exam; PSA, prostate-specific antigen.

^a^Subjects who provided written consent were coded as 1, while those who did not provide written consent were coded as 0.

In univariable analysis, participants providing written consent had a significantly lower risk of having PCa on biopsy. When stratified by cancer grade, the inverse associations between written consent status and cancer risk remained consistent for both low-grade and high-grade PCa. After adjusting for covariates, the inverse association between written consent status and overall PCa risk remained (OR = 0.41, *P* < .001), as well as for low-grade (OR = 0.54, *P* < .001) and high-grade PCa (OR = 0.33, *P* < .001) (Table 3).

**Table 3:** Univariable and multivariable logistic regression models assessing the association between written consent status and prostate cancer risk.

	**Overall prostate cancer**		**Low-grade prostate cancer** ^**a**^		**High-grade prostate cancer** ^**a**^	
**Variable**	**OR (95% CI)**	***P***	**OR (95% CI)**	***P***	**OR (95% CI)**	***P***
Univariable analysis						
Written consent						
Not provided	Ref		Ref		Ref	
Provided	0.61 (0.51-0.73)	<.001	0.71 (0.57-0.88)	.002	0.56 (0.46-0.68)	<.001
Multivariable analysis						
Written consent						
Not provided	Ref		Ref		Ref	
Provided	0.41 (0.31-0.54)	<.001	0.54 (0.39-0.73)	<.001	0.33 (0.24-0.44)	<.001
Age at biopsy (unit = 1 year)	1.04 (1.02-1.05)	<.001	1.01 (0.99-1.04)	.221	1.06 (1.03-1.08)	<.001
Race						
Non-Black	Ref		Ref		Ref	
Black	1.49 (1.18-1.88)	<.001	1.51 (1.15-1.99)	.003	1.44 (1.10-1.89)	.008
Year of biopsy (unit = 1 year)	1.08 (1.05-1.11)	<.001	1.06 (1.02-1.09)	<.001	1.11 (1.07-1.14)	<.001
PSA (ng/mL, log-transformed)	2.49 (2.08-2.98)	<.001	1.67 (1.36-2.03)	<.001	3.60 (2.91-4.45)	<.001
DRE						
Not suspicious for cancer	Ref		Ref		Ref	
Suspicious for cancer	2.16 (1.65-2.81)	<.001	1.29 (0.94-1.78)	.115	3.13 (2.33-4.20)	<.001
Prostate volume (cc, log-transformed)	0.19 (0.15-0.25)	<.001	0.27 (0.20-0.37)	<.001	0.14 (0.10-0.19)	<.001

Abbreviations: CI, confidence interval; DRE, digital rectal exam; OR, odds ratio; PSA, prostate-specific antigen.

^a^OR are vs no cancer.

There was a significant statistical interaction between written consent status and race for predicting risk of low-grade PCa (*P* = 0.017 for interaction), but not high-grade (*P* = 0.56 for interaction) or overall PCa (*P* = 0.079 for interaction). In stratified multivariable analysis by race, both Black and non-Black participants providing written consent had lower risk of overall PCa, though this only reached significance among non-Black patients. Similarly, patients providing written consent had a lower risk of high-grade PCa among both Black and non-Black patients. In contrast, written consent was associated with lower risk of low-grade PCa only among non-Black patients, but not among Black patients (Table 4).

**Table 4:** Univariable and multivariable logistic regression models assessing the association between written consent status and prostate cancer risk for subjects stratified by race**.**

**Race**	**Variable**	**Overall prostate cancer**		**Low-grade prostate cancer** ^a^	**High-grade prostate cancer** ^**a**^		
		**OR (95% CI)**	***P***	**OR (95% CI)**	***P***	**OR (95% CI)**	***P***
Univariable analysis							
Non-Black	Written consent						
	Not provided	Ref		Ref		Ref	
	Provided	0.50 (0.38-0.65)	<.001	0.49 (0.35-0.70)	<.001	0.50 (0.37-0.68)	<.001
Black	Written consent						
	Not provided	Ref		Ref		Ref	
	Provided	0.72 (0.57-0.91)	.005	0.91 (0.68-1.21)	.508	0.62 (0.48-0.80)	<.001
Multivariable analysis^b^							
Non-Black	Written consent						
	Not provided	Ref		Ref		Ref	
	Provided	0.53 (0.38-0.73)	<.001	0.52 (0.35-0.76)	<.001	0.54 (0.37-0.80)	.002
Black	Written consent						
	Not provided	Ref		Ref		Ref	
	Provided	0.78 (0.60-1.02)	.065	0.93 (0.69-1.26)	.634	0.65 (0.48-0.89)	.006

Abbreviations: CI, confidence interval; OR, odds ratio.

^a^OR are vs no cancer.

^b^Adjusted for age (continuous), year of biopsy (continuous), digital rectal exam (not suspicious for cancer/suspicious for cancer), prostate-specific antigen (continuous, log-transformed), and prostate volume (continuous, log-transformed).

## Discussion

Although chart-review studies are typically conducted using a waiver of written informed consent, not all IRBs will approve such a waiver under prospective enrollment. In cases where such a waiver is not allowed, the requirement of written consent could introduce selection bias leading to a less representative population in terms of baseline characteristics and clinical outcomes. In this study, we quantified this bias in a chart-review study using prospective enrollment with a waiver of written informed consent. By comparing participants based on whether they provided written consent to another separate minimal-risk prospective observational study protocol, we found that those providing written consent differed in many key aspects, but not by race. Importantly, patients providing written consent had a significantly lower risk of PCa. These findings suggest that participants who provided written consent might not accurately represent the entire eligible population, potentially due to a selection bias analogous to the “healthy worker effect.”^16^ The waiver of written informed consent with prospective enrollment allowed for data updates, the inclusion of new subjects, and extended follow-up without, as this analysis showed, introducing the selection bias created by requiring written consent. Thus, to best understand the entire population at risk and outcomes over time, IRBs should approve chart-review studies with prospective enrollment using the waiver of written informed consent.

Although many studies compared baseline characteristics between patients who did and did not provide written consent, few examined common primary outcomes in clinical research such as disease incidence, progression, and mortality. In our study, we found that patients who provided written consent had lower PCa risk in both unadjusted and adjusted analysis. In accordance with our findings, two previous studies reported that subjects who provided written consent for the use of their medical record had better overall survival compared to those who did not among patients diagnosed with breast cancer and brain arteriovenous malformations.^9^^,^^17^ Additionally, a secondary analysis of European Randomized Study of Screening for Prostate Cancer (ERSPC) found that those who participated had a lower all-cause mortality rate compared to the general target population.^18^ Taken together, these findings suggest that subjects who provide written consent may intrinsically differ from the broader eligible population in terms of not only demographics but also risk of the primary outcome. Interestingly, the direction of bias, including in our analysis, reported *better* outcomes among participants providing written consent. This pattern may arise because individuals who are healthier perceive themselves to be at lower disease risk or are more engaged in preventive care are more likely to participate in additional research activities, resulting in a selection bias similar to the “healthy worker effect.”^16^ As such, this provides yet more rationale for allowing prospective enrollment via a waiver of written informed consent in chart-review research.

We found significant differences in many demographic and clinical characteristics between subjects who did or did not provide written consent. For example, on multivariable analysis, older patients were much less likely to consent. This is relevant in that older age is a significant risk factor for PCa.^19^ This finding aligns with a previous study on biobank participation among African American PCa survivors, which reported that participants were less likely to be older than 64 years compared to nonparticipants.^20^ Similarly, another secondary analyses of ERSPC also found that consenters were younger compared to nonconsenters.^21^^,^^22^ According to a previous study, older individuals are less likely to visit doctors and tend to be less proactive about their health, which could lead to their lower participation in clinical studies.^23^ Given written consent tends to skew the population to the younger side, when performing chart-review studies without the waiver of written informed consent, researchers should recognize that the study population is only a subset of the entire patients at risk and should be cautious in interpreting the results.

Black race is another significant risk factor for PCa incidence and mortality.^19^ Indeed, in our study, Black race was a significant risk factor for both high- and low-grade PCa at biopsy. As such, understanding potential selection by race is crucial. It is well-known that Black patients are underrepresented in clinical cancer research.^24^^,^^25^ Fortunately, we found Black patients were equally likely to provide written consent in both unadjusted and adjusted analysis. Consistent with this, a prior meta-analysis across 35 cancer clinical trials also found Black patients participated at similar rates as White patients.^26^ Similarly, another study concluded that willingness to participate in PCa clinical trials does not differ significantly by race.^27^ Therefore, the underrepresentation of Black patients in clinical trials might be more closely related to racial disparities in access to medical centers conducting these studies, such as the geographic distance from such centers, rather than differences in willingness of participation.^28^^,^^29^ As such, to improve the disproportionately low number of Black patients on PCa trials, we need to not necessarily improve enrollment rates among Black patients but rather open sites that have abundant Black patients such that more can participate. As sites with a high percentage of Black patients are often under-resourced, concerted efforts are needed to support research infrastructure at sites that care for a high percentage of Black patients including, but not limited to, intercity and VA hospitals.

Although race was not directly associated with written consent status, it did modify the association between providing written consent and lower risk of low-grade PCa. Specifically, people who provided written consent had lower risk of high-grade PCa regardless of race, but the lower risk of low-grade PCa was only seen among non-Black men. The lower risk of high-grade PCa may be explained by selection bias, as noted above, in that people who provide written consent to studies tend to be healthier and have better outcomes. However, the reason this was only seen for low-grade PCa among non-Black patients is not clear. As participants were asked to provide written consent before biopsy results were available, knowledge of PCa status or grade is unlikely to explain this finding. Given this unexpected finding without an obvious explanation, this requires confirmation in future studies. If confirmed, then future research should aim to understand the reasons for this association.

Our study identified significant differences among individuals who did and did not provide written consent, suggesting that to mitigate selection bias, chart-review studies should be allowed to prospectively recruit participants using a waiver of written informed consent. However, whether IRBs will approve protocols with prospective enrollment under a waiver of written informed consent remains highly unpredictable.^4^ This uncertainty stems from the regulatory requirements for granting a waiver of written informed consent. In addition to the minimal-risk nature, IRBs must also determine that “the study could not practicably be carried out without the requested waiver,”^1^ a judgment that varies across IRBs. Furthermore, as it is theoretically possible to obtain written consent at the time of prospective enrollment, IRBs may take a conservative approach in granting such approvals. However, prior studies have suggested a more flexible interpretation of impracticality beyond clear cases such as unreachability of participants in retrospective analyses.^30^^-^^32^ Specifically, when the requirement for written informed consent introduces significant selection bias that compromises study validity, as demonstrated in our findings, the impracticality might refer to inability to achieve the study objectives even if obtaining written consent is technically feasible. Although consent and study outcomes are causally independent, requiring written consent may introduce inherent differences between participants that could be mitigated by a waiver of consent. Therefore, if the evidence of selection bias due to requiring written informed consent is presented, the waiver of written informed consent should be permitted for prospective enrollment in chart-review studies to preserve scientific integrity.

Our study should be interpreted in the context of several limitations. First, while we identified participants by the chart-review only protocol, written consent status was determined by another separate protocol requiring blood collection and questionnaire completion. Had the written consent only been for reviewing medical records, the number of patients providing written consent might have been higher and perhaps the selection bias would have been less. However, as a single blood draw and completing questionnaires are usually considered as minimal-risk research activities, we expect that few patients refused providing written consent due to concerns about these additional requirements. Furthermore, 52% of participants in our study provided written consent, which is comparable to a median participation rate of 53% in the systematic review of 17 studies where participants were approached solely for the use of their medical records.^8^ However, this comparison should be interpreted cautiously, as studies included in the systematic review varied in design and may have involved additional participant-facing procedures beyond medical record review. Therefore, while the requirement for a single blood draw and questionnaires may not have substantially reduced participation in our study, its independent impact on consent rates cannot be fully disentangled from other study- and population-level factors. Second, we did not adjust for some potential confounders, such as socioeconomic status, diet, and exercise, as these data were only collected through questionnaires and thus unavailable for patients who did not provide written consent. However, we adjusted for the most well-established confounders for PCa risk, including race, PSA, and DRE, which supports the robustness of our conclusions. Third, a small number of participants undergoing prostate biopsies at the DVAHCS were not approached for enrollment of the blood collection protocol due to scheduling issues, which would have been included in the no written consent group. Finally, as our study was conducted within the VA healthcare system, the generalizability of our findings to other healthcare settings needs additional investigation.

In conclusion, we identified patients for a chart-review study using prospective enrollment with a waiver of written informed consent and found significantly different baseline characteristics and lower PCa risk, but no differences by race among those who provided written consent to another separate minimal-risk prospective observational study protocol compared to those who did not. These findings strongly support that the requirement for written consent for chart-review studies results in a selection bias among the study cohort and underscores the importance of allowing prospective enrollment with a waiver of written informed consent for chart-review studies.
